# Meeting of the Alumni Association of Buffalo Medical College

**Published:** 1879-03

**Authors:** 


### MEETING OF THE ALUMNI.

The fifth annual meeting of the Alumni Association of the Medical Department of the University of Buffalo opened shortly before eleven o'clock, at the College building.

The Association was called to order by Dr. W. C. Phelps, who introduced the President-elect, Dr. M. Baker of Warsaw, who returned his thanks for the honor conferred upon him in his election, and then introduced Prof. James P. White, M. D., who, in consequence of the inability of Prof. Moore to reach the city, delivered the address of welcome.

On motion of Dr. Briggs, the following committees were appointed:

*On President's Address*—Doctors J. S. Smith and W. C. Phelps.

*Nomination of Officers*—Doctors D. W. Harrington, W. H. Slacer and A. R. Davidson.

At three o'clock the Alumni again convened, and after listening to the reading of two very interesting papers and the address of Dr. Baker, the retiring President, the Association proceeded to elect the following officers for the ensuing year:

President—C. H. Richmond, Livonia, N. Y.
First Vice President—Wm. C Phelps, Buffalo.
Second Vice President—T. M. Johnson, Buffalo.
Third Vice President—Eugene Smith, Detroit.
Fourth Vice President—Henry Lapp, Clarence.
Fifth Vice President—R. Y. Charles, Rushford, N. Y.
Secretary—A. M. Barker, Buffalo.
Trustee—Prof. James P. White, Buffalo.
Executive Committee—A. H. Briggs, W. H. Slacer, S. G. Dorr, James P. White, *ex officio*, and C. H. Richmond, *ex officio*.

Dr. J. S. Trowbridge, of this city, Dr. J. B. Andrews, of Utica, and Dr. H. C. Tompkins, of Tompkinsville, N. Y., were elected honorary members.

Dr. Hall then gave notice that at the next annual meeting he should present an amendment to the constitution, providing that the meetings of the Association be held once in two years.

Dr. Sloan offered an amendment to the constitution, providing that the election of officers be by ballot.

Under the rule, this was laid over for one year.

There being no further business, the Association adjourned.

At the conclusion of the exercises in St. James Hall, the Faculty, Curators and Alumni, with their friends, adjourned to the Tifft House, where the Annual Alumni Banquet was spread.

